# The economic burden of cervical cancer in Eswatini: Societal perspective

**DOI:** 10.1371/journal.pone.0250113

**Published:** 2021-04-15

**Authors:** Cebisile Ngcamphalala, Ellinor Östensson, Themba G. Ginindza

**Affiliations:** 1 Discipline of Public Health Medicine, School of Nursing and Public Health, University of KwaZulu-Natal, Durban, South Africa; 2 Department of Women’s and Children’s Health, Karolinska Institutet, Stockholm, Sweden; 3 Department of Medical Epidemiology and Biostatistics, Karolinska Institutet, Stockholm, Sweden; National Center for Global Health and Medicine, JAPAN

## Abstract

**Background:**

Cervical cancer imposes considerable economic burden on societies and individuals. There is lack of evidence regarding this from the developing world and particularly from sub-Saharan Africa. Therefore, the study aimed to estimate the societal costs of cervical cancer in Eswatini.

**Materials and methods:**

The cost of illness study (CoI) was applied using national specific clinical and registry data from hospitals, registries and reports to determine the prevalence of cervical intraepithelial neoplasia (CIN) and cervical cancer in Eswatini in 2018. Cost data included direct medical costs (health care utilization in inpatient and outpatient care), direct non-medical costs (patient costs for traveling) and indirect costs based on productivity loss due to morbidity (patient time during diagnosis and treatment) and premature mortality.

**Results:**

The estimated total annual cost for cervical cancer was $19 million (ranging between $14 million and $24 million estimated with lower and upper bounds). Direct cost represented the majority of the costs at 72% ($13.7 million) out of which total pre-cancerous treatment costs accounted for 0.7% ($94,161). The management of invasive cervical cancer was the main cost driver with costs attributable to treatment for FIGO III and FIGO IV representing $1.7 million and $8.7 million respectively. Indirect costs contributed 27% ($5.3 million) out of which productivity loss due to premature mortality represented the majority at 67% ($3.5 million).

**Conclusion:**

The economic burden of cervical cancer in Eswatini is substantial. National public health prevention strategies with prophylactic HPV vaccine and screening for cervical lesions should therefore be prioritized to limit the extensive costs associated with cervical cancer.

## Introduction

Among other cancers, worldwide, cancer cases attributed to the Human Papilloma virus (HPV) are estimated at 4.5% (630 000) [1]. Evidence shows that high risk HPV types cause almost 100% of cervical cancer cases [1–3]. The burden of cervical cancer is compounded by the high prevalence of HIV infection, particularly in settings with limited access to the HPV vaccine [4]. The life time risk for cervical cancer in HIV positive women is higher (3.3%) than that in HIV negative women which stands at 2.3% [5].

Cervical cancer is the 4^th^ most frequent female cancer and the 4^th^ leading cause of cancer mortality worldwide [6]. However, the disease burden is not equally distributed across countries. Low- and middle-income countries (LMIC) report high mortalities compared to high-income countries (HIC) [6, 7]. The age standardized (world) incidence rates (per 100,000) of cervical cancer cases were among the highest in sub-Saharan Africa (≥26) in 2018 [6]. Cervical cancer imposes a considerable economic burden on society and individuals [6, 8]. However, there is limited knowledge of the economic burden of cervical cancer in low-income countries (LICs) and particularly in sub-Saharan Africa [9]. Most studies estimate the economic burden either for multi-country or country-specific aggregate data of multiple cancers [8–12].

Understanding the human and economic burden of the disease is important for public health policy-makers to make planning and budget decisions [12]. Findings from CoI studies provide essential information on disease-specific costs and data for further economic evaluations in view of informed policy decisions [13–15]. With evidence of HPV vaccination preventing up to 90% of diseases attributed to this virus the question of how to prioritize and allocate resources to prevent cervical cancer is essential. We therefore aim to investigate the societal cost of cervical cancer in Eswatini to contribute to further economic evaluations important for informed policy-making.

Our previous cost analysis conducted in Eswatini estimated direct medical costs associated with Human Papillomavirus (HPV) related cervical cancer diseases at $16 million [14]. This was the first study to demonstrate the magnitude of the economic burden resulting from cervical cancer. Whilst the study demonstrated the significant economic burden of cervical dysplasia and invasive cancer, it only considered the providers’ perspective. Indirect costs tend to contribute a significant proportion of the total economic burden/or societal cost of HPV-related diseases [16] and are therefore included in our present study.

## Materials and methods

### Method of costing

From a societal perspective, we performed a Cost of Illness (CoI) study, investigating costs incurred during the intervention both by the provider and the patients [15]. CoI is the common methodology for estimating specific disease-associated costs [13].

‘Prevalence-based’ and ‘Incidence-based’ methods are the most common approaches to conducting CoI studies. We used a prevalence-based approach which considers costs of all disease cases in a geographical area in a given time period [13]. We employed both top-down and bottom-up costing approaches [15, 17]. A top-down costing approach was used to estimate the costs associated with health care services whilst a bottom-up costing approach (micro-costing) was used to determine costs associated with primary care.

### Costs

A societal perspective, including direct medical, direct non-medical and indirect costs (monetary value of productivity loss due to morbidity and mortality), was used to estimate the economic burden associated with cervical cancer in Eswatini. Direct medical costs were divided into recurrent costs and capital costs [15]. Recurrent costs included personnel, travel, consumables (including medical supplies), administration, utilities and overheads. Capital costs consisted mainly of equipment, buildings, vehicles and everything that has a useful life of more than one year and an equivalent value of $50. All costs are presented in US Dollars using the 2018 average exchange rate of1 USD ($) = 14.5 Eswatini lilangeni (SZL), the Eswatini currency.

#### Direct medical costs

To estimate the total direct medical costs, we estimated the average cost of screening and managing cervical lesions and cervical cancer. The average cost for each treatment was then multiplied by the number of patients treated corresponding. The number of women screened by Pap smear were obtained from the Sexual Reproductive Health (SRH) annual reports and prices were based on market or private sources obtained through the Phalala Fund [18] (Table 1). Data on the cytological results including normal or abnormal results and inadequate cytological results (result could not be determined) were not available. We estimated the burden of ASCUS-H or HSILCIN2/CIN using the treatment information provided in the SRH 2018 report [19]. Laboratory costs including cytological testing, follow-up biopsy as well as physician assessment of abnormal results were obtained from the private sector and market pricing [18]. The number of Visual Inspection with Acetic Acid (VIA) screened and VIA positives were obtained from the SRH report. Treatment costs were estimated as per the procedures provided in the Eswatini Standardized Cancer Care and Guidelines [20]. Variables were adapted from a previous study conducted in Eswatini [14]. The total number of women treated with loop electrosurgical excision procedure (LEEP), or total abdominal hysterectomy (TAH) and Cryotherapy was obtained from the SRH 2018 report.

**Table 1:** Data variables and source for costs regarding screening, management and treatment of cervical lesions and cervical cancer.

Data	Data source	Price source
Estimated number of cases in 2018 = 268	Swaziland National Cancer Registry (SNCR), Ministry of Health, Report on Cases of Cancers in Swaziland (2018)	
**Screening**		
VIA screening	Sexual and Reproductive Health Annual Program Report, 2018 Health Management Information System (HMIS)	Market price
Pap smear/Cytology	Eswatini Health Laboratory Service (EHLS)	Private hospital
**Lesion and cancer diagnosis and treatment**		
Treatment with LEEP	Sexual and Reproductive Health Annual Program Report, 2018 Eswatini Health Laboratory Service (EHLS)	Market price
Treatment with cryotherapy (for VIA positives)	Sexual and Reproductive Health Annual Program Report, 2018	Market price
Follow-up Pap smear of LSIL /CIN1	Private hospital, gynecologist	Market price/private hospital
Treatment of AGC; ASCUS-H; HSIL-/CIN2/CIN3 and FIGO 1 with hysterectomy	Private hospital, Phalala Fund	Market price/private hospital
Treatment of FIGO II-IV with radiotherapy and chemotherapy + Biopsy	Phalala Fund based on SA hospitals fees	Market price

Information to estimate treatment costs associated with cervical cancer was obtained from the Phalala Fund through review of medical records. Similarly, treatment variables were adapted from a previous study [14] and information from expert interviews, these included medical practitioners from private facility (The Clinic Group—Mbabane) and public facility (Mbabane Government Hospital—Chemotherapy Unit).

Actual costs of the variables were estimated at market price based on private charges [18]. The country lacks official data on the proportion of women treated for different International Federation of Gynecology and Obstetrics (FIGO) stages of cancer. Provided proportions were based on the empirical number of patients diagnosed and treated according to the Cancer Registry annual report, Table 1. We assumed that the reported number of women with cervical cancer were clinically staged according to FIGO staging procedures and were treated as per the Eswatini Standardized Cancer Care and Guidelines. In addition to clinical assessment of patients diagnosed, other investigations included biopsy, X-ray, CT-computed tomography scan which are currently available in private hospitals. The Phalala Fund kept data for all patients diagnosed with invasive cancer receiving treatment in and outside the country. Data variables and cost for screening, management and treatment of cervical lesions and cervical cancer were based on sources presented in Table 1.

#### Direct non-medical costs

A cross-sectional study was conducted on nineteen (19) patients with cervical cancer attending treatment follow-up at the Mbabane Government Referral Hospital and we collected data on the amount spent on transport and patient time costs (monetary value of the time spent away from work as a result of seeking cancer management).

We estimated transportation costs (including return) for women receiving follow-up treatment, specifically chemotherapy at the Chemotherapy (outpatient) Unit of the Mbabane Government Referral Hospital. We used Eswatini Standardized Cancer Care and Guidelines and interviewed experts to obtain the average number of visits for each woman in a year. A majority of the patients indicated that they needed to have a companion or a relative whenever they visited the hospital. We therefore doubled transport costs to account for the companion’s transport costs.

#### Indirect costs

The number of cervical cancer deaths for 2018 was obtained from the national Cancer Registry.

*Morbidity*. Employing the human capital approach [21], the time-related gross earnings were estimated. Indirect costs related to patient time taken for seeking health care was estimated. To compute patient time costs due to prevention and treatment for cervical cancer we estimated average monthly income from gross annual earnings. Adapting a methodology described elsewhere [22], we estimated patient time by screening methodology, diagnosis, treatment for CIN, staging and treatment by FIGO stage.

*Patient time by screening methodology*, *diagnosis*, *treatment for CIN*. Information on waiting time and procedure was gathered from experts in the field, mainly from private hospitals. The Eswatini Standardized Cancer Care and Guidelines states that follow-up care includes quarterly gynecological reviews with colposcopy and biopsy in the first year of treatment [20]. The study assumed 20 full-time working days of 8 hours per month. Following recommendations for health economic evaluations, the average annual general gross earnings rate for all working individuals of both genders was used [15, 23]. Cost per work day ($11.2) and work hour ($1.4) was estimated. Then we multiplied the total procedure and wait time per parameter in the prevention, management and treatment of CIN with the corresponding number of people who received the care.

*Patient time for staging*, *treatment and follow-up by FIGO stage*. Staging and treatment, particularly radiotherapy, is conducted in South Africa. Using data from literature [22] and information obtained from the Phalala Fund, we estimated that patient staging is one week on average (40 working hours, translating to $56 per person). To compute productivity loss, we multiplied the value of time lost with the number of women diagnosed with cervical cancer in 2018 (268), Table 1. Similarly, to establish productivity loss as a result of invasive cancer treatment, we established the treatment time including follow-up time in weeks by FIGO stage resulting to FIGO Ia1-Ib1 (9), FIGO Ib2-II (17), FIGO (III) and FIGO IV (42). The Eswatini Standardized Cancer Care and Guidelines states that follow-up care includes a gynecological examination including a Pap smear every 3 months for the first 2 years after completing treatment.

*Mortality costs*. Calculating mortality costs required three parameters, that is the number of deaths, years of potential productive life lost (YPPLL) and monetary value of productivity lost [24]. The human capital approach equates productivity lost to an individual wage rate and is based on the assumption that an individual continues to produce output over a working life time which is cut short by premature death [24]. Data from the national cancer registry was used to estimate the number of cervical cancer related deaths for 2018. To estimate the YPPLL due to premature cervical cancer related death, which is defined as the remaining life expectancy at the age of death. Average expected years of potential productive life lost for cervical cancer age-group-specific deaths was estimated assuming labor participation ages of Eswatini (18–60 years). Furthermore, the study used full employment rate in the cervical cancer patients and annual average earnings. Average YPPLL was multiplied by average annual earnings. According to health economic recommendations, future costs were discounted at 3% and 5% [15, 23]. To account for uncertainty, we based our calculations on both the assumptions that the women lived until the end of the year and that the women died in the middle of the year.

### Estimation of annual costs

The aggregate cost of prevention, management and treatment of cervical intraepithelial neoplasia and cervical cancer in 2018 was computed. All costs were reported in 2018 US dollars (average exchange rate: $1 = SZL14.5).

### Sensitivity analysis

Using methodology adapted from previous studies [11, 14, 22, 25], we performed sensitivity analysis using ±25% to account for uncertainties in the cost estimation and unrecorded cases by the facilities.

### Ethics approval and consent to participate

The study was approved by the National Health Research Review Board (NHRRB) of Eswatini (FWA 00026661/IRB 00011253) and the University of KwaZulu-Natal Biomedical Research Ethics Committee (BE 059/19). Both ethics committees approved the data collection tool and written informed consent form, which was obtained from all the participants prior to participating in the study. All women aged between 18 years and above receiving chemotherapy care at the Mbabane Government Referral Hospital—Chemotherapy Unit were considered eligible for the study.

## Results

### Prevention, management and treatment of cervical intraepithelial neoplasia (CIN)

Cost data was collected to estimate the economic burden associated with prevention, management and treatment of cervical intraepithelial neoplasia. Parameters and variables included in the study are presented in Table 2.

**Table 2:** Costs for prevention, management and treatment for cervical lesions expressed in 2018 USD average exchange rate of $1 = SZL14.5.

Parameter	Variables included in the cost	Average (2018) USD
Pap smear screens	Examination table with foot supports, examination light, speculum, examination gloves, cervical spatula and cytobrush glass slide and fixative, 1 sheet	48
Total VIA screened	Nurse’s time, examination table, speculum, light source (halogen torch or flashlight), instrument tray, 3 cotton swabs, examination gloves, 20mls of 3% to 5% acetic acid (white table vinegar) solution	38
Biopsy	Colposcope: Variable fixed power or zoom lens (3× to 7× low power to 15× to 40× high power), biopsy forceps, endocervical curette (Kevorkian curette, no basket), endocervical speculum (Kogan, both narrow and wide types), ring forceps, Pap smear materials, vaginal speculums, full-strength Lugol’s iodine solution, Monsel’s solution (ferric subsulfate), 1 ml, acetic acid solution 3% to 5% (white vinegar; 4–6 oz or 120–180 ml), cotton- or rayon-tipped swabs (8–10), junior Scopettes/OB-GYN applicators (6–10), 4 × 4 gauze, urine or sputum cups for vinegar, vaginal side wall retractor, underpads ("chuck pads") (17 × 24 inch), cotton balls (15–20), power (electricity), doctor’ss time, Lab cost	95
LEEP	Usage of all equipment/instruments, linen and consumables: 1 packet of cotton balls, 1 packet gauze, antiseptic solutions—Savlon ±50cc, floor cleaning solution-sonic 1 bucket, specimen bottle, formalin 10cc, Cidex solution 5L, vinegar solution, anesthesia, doctor’s and nurse’s time	512
Cryotherapy	Usage of cryotherapy machine and equipment, nitrous oxide, sanitary pad, detergents (95% alcohol, water, sodium chloride solution), nurse’s time, antibiotics treatment: Amoxylin/Ciproflaxin 500mg BID, Flagyl 400mg tid 7/7 days OR: Panado 1gm TDS and Diclofenac 50mg TDS 3/7 days	38
Follow-up procedure of LSIL	With Pap smear and antibiotics	56
Treatment of HSIL	With hysterectomy during anesthesia: usage of equipment/instruments, linen, 5 swabs abdominal packs, 20 gauges, sutures– 4 vicryl No.2 and 2 vicryl No.1, anesthesia, doctor’s and nurse’s time	998
		**1785**

### Treatment of cervical cancer

The breakdown for staging and treatment variables for FIGO I- IV (total number of sessions per stage) is presented in Table 3. The costs for staging, management and treatment of cervical cancer per FIGO stage including cost breakdown of variables resulted in a cost of $2,445 for FIGO Ia1-Ib1, $32,870 for FIGO Ib2-II, and $32,666 and $54,421 for FIGO III and IV respectively, Table 4.

**Table 3:** Breakdown for staging and treatment variables for FIGO I-IV.

Staging and treatment variables	IA1-IB1	1B2-II	III	1V
Transport including from Eswatini and between treatment sites	0	1	1	1
Lodging including meals	0	42	42	42
Hospital admission for early stage treatment	1	0	0	0
Hospital admission in step down facility	0	1	1	1
Radical hysterectomy with anesthesia	1	0	0	0
Clinical examination with colposcopy under anesthesia	1	1	1	1
CT scan planning	0	1	1	1
Brachytherapy treatment	0	1	1	0
Radiotherapy treatment		35	35	35
Follow-up during brachytherapy & radiotherapy	0	2	2	2
Chemotherapy	0	7	7	7
Follow-up during chemotherapy	0	2	3	1
Follow-up after treatment	1	12	12	12
MRI		1	1	1
Nurses’ and drivers’ allowance	0	2	2	2

**Table 4:** Costs for staging, management and treatment of cervical cancer per FIGO stage I-IV expressed in 2018 USD average exchange rate of $1 = SZL14.5.

Breakdown for staging and treatment variables for FIGO I-IV					
Staging and treatment variables	Cost per unit ($)	IA1-IB1	1B2-II	III	1V
Transport including from Eswatini and between treatment sites	1,771	0	1,771	1,771	1,771
Lodging including meals	55	0	2,310	2,310	2,310
Hospital admission for early stage treatment	1,207	1,207	0	0	0
Hospital admission in step down facility	105	0	105	105	105
Radical hysterectomy with anesthesia	1,034	1,034	0	0	0
Clinical examination with colposcopy under anesthesia	362	0	362	362	362
CT planning scan	235	0	235	235	235
Brachytherapy treatment	4,969	0	4,969	4,969	0
Radiotherapy treatment	402	0	14,070	14,070	14,070
Follow-up during brachytherapy & radiotherapy	204	0	408	408	408
Chemotherapy	527	0	3,689	3,689	3,689
Follow-up during chemotherapy	204	0	612	408	204
Follow-up after treatment	204	204	2,448	2,448	29,376
MRI	965	0	965	965	965
Nurses’ and drivers’ allowance	463	0	926	926	926
TOTAL	12,707	2,445	32,870	32,666	54,421

The distribution of the cervical cancer cases was as follows: FIGO Ia1-Ib1 (13), FIGO Ib2-II (40), FIGO III (54) and FIGO IV (161) (Table 5). Staging was conducted outside the country, mainly in South Africa (SA), and involved clinical examination with cystoscopy under anesthesia. FIGO stage Ia1-Ib1 treatment involved hysterectomy, a procedure conducted locally. Treatment for FIGO stage Ib2-IV involved brachytherapy, radiotherapy, chemotherapy and blood tests conducted in SA over 42 days. Brachytherapy use was limited to metastatic cancer, hence the variation in cost distribution in FIGO stages III and IV. Additional costs incurred as a result of seeking treatment in SA included transport, lodging and accompanying staff allowance. Applying the staging, treatment and follow-up procedures, the annual estimated cost for cervical cancer treatment was $11,8 million (ranging between $8,9 million—$14,8 million) (Table 5). The estimated annual direct costs for prevention, management, and treatment of CIN and invasive cervical cancer was $13,7 million, out of which pre-cancerous treatment costs accounted for $94,161. When accounting for uncertainty in the number of women that were eventually screened versus those not screened (sensitivity analysis (±25)), the total annual cost ranged from $10,3 million to $17,1 million (Table 5).

**Table 5:** Annual direct (health care costs) cost estimation for prevention, management and treatment of cervical cancer lesions and cancer expressed in $ for 2018.

	Prevalence 2018	Cost per item	Base case cost	Range	
Parameter	Number	Average cost (2018)	Base costs (2018)	Lower (-25%)	Higher (+25)
Screening	22,345				
Pap smear	4,248	48	203,904	152,928	254,880
Total VIA screened	18,097	38	687,686	515,765	859,608
Biopsy	387	95	36,765	27,574	45,956
Total screening costs			928,355	696,266	1,160,444
**Diagnosed cervical lesion**					
Treatment with LEEP	527	512	269,824	202,368	337,280
VIA positive treated with cryotherapy	515	38	19,570	14,678	24,463
Follow-up of LSIL with Pap smear 3 months later	10,132	56	567,392	425,544	709,240
Treatment of HSIL with hysterectomy under anesthesia	85	998	84,830	63,623	106,038
Total pre-cancerous treatment costs			941,616	706,212	1,177,020
**Diagnosed with cervical cancer**					
FIGO IA-IBI	13	2,445	31,785	23,839	39,731
FIGO IB2-II	40	32,870	1,314,800	986,100	1,643,500
FIGO III	54	32,666	1,763,964	1,322,973	2,204,955
FIGO IV	161	54,421	8,761,781	6,571,336	10,952,226
Total cost for treating invasive cancer			11,872,330	8,904,248	14,840,413
**Total direct costs**		37,100	13,742,301	10,306,726	17,177,876

### Direct non-medical costs

A total of 268 women were diagnosed with invasive cancer in 2018. The mean transport cost per visit including return was estimated at $10. With 12 visits annually including a relative companion in all the visits, the annual transport costs was estimated at $0,06 million (ranging between $0,05–$0,08 million).

### Indirect costs

#### Patient time costs

The total cost for patient time spent by screening methodology, diagnosis and treatment of CIN was estimated at $1,2 million (Table 6). We further estimated patient time for staging, cancer treatment and follow-up per FIGO stage. The total costs estimated stood at $0,5 million (Table 7).

**Table 6:** Assumed patient time spent and productivity lost by screening methodology, diagnosis and treatment of CIN.

Screening strategy	Procedure time (minutes)	Wait time (minutes)	Total time (minutes)	Total times (hours)	Productivity loss ($)	Productivity loss by number of patients seen in 2018 ($)
VIA screening	60	20	100	1,7	2,3	42,226
Pap smear/cytology	20	20	40	0,7	0,9	3,965
**Treatment for the lesions**						
Cryotherapy	60	20	80	1,3	1,9	961
LEEP	60	20	80	1,3	1,9	984
Follow-up after treatment for CIN (four gynecological reviews including cytology tests)	60	40	100	80	112	113,478
Total	260	120	400	85	119	1,182,920

**Table 7:** Patient time for staging, cancer treatment and follow-up by FIGO stage.

FIGO stage	Treatment time including staging (weeks)	Valuing treatment time (weekly income $56)	Follow-up (hours)	Valuing follow-up time ($)	Productivity loss (sum of the value for treatment time and follow-up time) ($)	Productivity loss by number of women corresponding per FIGO stage in 2018 ($)
Ia1-Ib1	9	504	15	22	5,254	6,831
Ib2-II	17	952	15	22	973	38,938
III	17	952	15	22	973	52,567
IV	42	2,408	15	22	2,429	391,144
Total	85	4,816	60	86	4,901	489,481

#### Mortality

According to the national cancer registry, there were 296 cancer-related deaths in 2018 [26], out of which 141 were attributed to cervical cancer. Of these 141 deaths, 52 were of women above the labor participation age of Eswatini (60 years). Only deaths of women aged between 18–60 years were included in our calculation of potential productive life lost due to premature cervical cancer related mortality. The total costs of premature deaths due to cervical cancer at 3% discount rate was estimated at $1.9 million and at 5% discount rate $1.3 million (Table 8). Varying the total costs of premature deaths due to cervical cancer by half (0.5) at 3% discount rate, the total costs was estimated at $1.3 million and at 5% discount rate $1.0 million (Table 8).

**Table 8:** Estimating mortality costs.

Mortality cost for cervical cancer varied for 1 and 0.5 years							
Age group	Lost YPPLL	Number of deaths	Average annual income ($)	Mortality cost at 3% discount rate ($) (assuming women lived for 1 year)	Mortality cost at 5% discount rate ($) (assuming women lived for 1 whole year)	Mortality cost at 3% discount rate ($) (assuming women lived for 0.5 years)	Mortality cost at 3% discount rate ($) (assuming women lived for 0.5 years)
23–27	35	2	2,690	64,843	31,274	55,249	38,369
28–32	30	6	2,690	194,168	103,928	153,310	112,163
33–37	25	16	2,690	502,465	298,471	367,645	283,353
38–42	20	15	2,690	438,842	289,298	297,551	241,590
43–47	15	15	2,690	383,275	280,407	240,820	205,983
48–52	10	7	2,690	138,857	112,742	80,850	72,851
53–57	5	13	2,690	150,150	135,296	81,015	76,903
58–60	1	5	2,690	13,047	12,778	6,623	6,555
**Totals**	**1,315**	**79**	**2,690**	**1,885,646**	**1,264,193**	**1,283,063**	**1,037,768**
Average annual gross income = $2,690							

### Total annual cost

The total annual cost for cervical cancer was estimated at $19 million (between $14 million and $24 million estimated with lower and upper bounds). Direct costs constituted a majority of the costs at 72%, ($13,7 million) (Table 9). Management for invasive cancer stage was the main cost driver with costs attributable to treatment for FIGO III and FIGO IV representing $1,7 million and $8,7 million respectively. Indirect costs contributed 27% ($5,3 million) out of which productivity loss due to premature mortality represented 67% ($3,5 million).

**Table 9:** Total annual cost estimation for cervical cancer lesions and invasive cancer (direct, direct non-medical and indirect costs) in 2018.

	Prevalence 2018	Cost per item	Base case cost	Range	
Parameter	Number	Average cost (2018)	Base costs (2018)	Lower (-25%)	Higher (±25)
**Direct costs (health care costs)**					
Screening	22,345				
Pap smear	4,248	48	203,904	152,928	254,880
Total VIA screened	18,097	38	687,686	515,765	859,608
Biopsy	387	95	36,765	27,574	45,956
**Total**			**928,355**	**696,266**	**1,160,444**
**Diagnosed cervical lesion**					
Treatment with LEEP	527	512	269,824	202,368	337,280
VIA positive treated with cryotherapy	515	38	19,570	14,678	24,463
Follow-up of LSIL with Pap smear 3 months later	10,132	56	567,392	425,544	709,240
Treatment of HSIL with hysterectomy under anesthesia	85	998	84,830	63,623	106,038
**Total**			**941,616**	**706,212**	**1,177,020**
**Diagnosed with cervical cancer**					
FIGO IA-IBI	13	2,445	31,785	23,839	39,731
FIGO IB2-II	40	32,870	1,314,800	986,100	1,643,500
FIGO III	54	32,666	1,763,964	1,322,973	2,204,955
FIGO IV	161	54,421	8,761,781	6,571,336	10,952,226
Total			11,872,330	8,904,248	14,840,413
**Total direct costs**		**37,100**	**13,742,301**	**10,306,726**	**17,177,876**
**Direct non-medical costs**					
Patient transport costs from seeking health care assuming that in all the visits the patient had at least one companion (268 patients)	536	120	64,320	48,240	80,400
**Indirect costs**					
Patient time off work from seeking health care by screening methodology, diagnosis, treatment for CIN, staging and treatment by FIGO stage	268	6,240	1,672,401	1,254,301	2,090,502
Mortality (premature deaths)	1,315	2,690	3,537,350	2,653,013	4,421,688
Total			5,274,071	3,955,553	6,592,589
**Total**		**9,050**	**19,016,372**	**14,262,279**	**23,770,465**

## Discussion

This is the first study in Eswatini to evaluate the societal costs of prevention and treatment of cervical intraepithelial neoplasia and invasive cancer. The study estimated that the annual societal cost of cervical cancer in 2018 was $18,9 million, ranging from $14 million to $24 million. Costs for health care and productivity loss represented 72% ($13,7 million) and 27% ($5,2 million) of the total costs respectively. The main cost driver was health care cost for treatment of invasive cervical cancer with this cost rising steadily with late disease treatment, FIGO stages I ($0,4million), II ($1,6million), III and IV ($2,2million and $10,1 million respectively).

This findings are consistent with those of previous studies whose findings suggest that costs of managing cervical cancer vary by cancer stage at diagnosis and that advanced cervical cancer (stages III and IV) poses the greatest costs [27–30]. Liu et al. reported significant costs of cervical cancer in Canada and, according to these researchers, the average incremental costs increased with treatment phase (pre-diagnosis ($362), initial phase ($15,722), continuing phase ($3,924) and $52,539 in the terminal phase) [28]. Similarly, a study assessing the costs of cancer care on a treatment continuum for 10 malignancies including cervical cancer concluded that stage III cancers posed the greatest annual cost burden for most cancer types [31]. A systematic review assessing the cost of cancer along the treatment phases also concluded that costs vary by cancer stage [32]. In contrast, Patel et al. [33] reported that screening constituted the highest proportion of the annual cost in 4 European countries, namely Belgium $23 million (€22.6 million); Finland $35 million (€30.7 million), Poland $58 million (€49.8 million) and Sweden $58 million (€49.8). The current findings corroborate the conclusions by other studies, asserting that countries with well-established screening programs incur higher expenses on prevention measures compared to developing countries with less organized screening intervention [14, 34]. This evidence is reasonable and seems to reflect how cancer programming is organized in HICs versus LMICs. The consumption of screening services is expected to be higher in HIC countries due to service availability compared to LICs. However, more recent studies from both worlds are necessary to better understand such dynamics across countries.

Our previous cost analysis conducted in Eswatini showed that treatment of high grade cervical lesions and invasive cancer constituted most of the costs, accounting for about 80% ($12.6 million) of the total costs ($16 million) [14]. Similarly, our previous study conducted in Sweden found that direct costs accounted for 76% of the total costs for which costs of prevention, management and treatment of CIN were the main cost drivers [11]. Whilst direct costs contribute significantly to the economic burden of cervical cancer, our findings demonstrated that productivity loss as a result of premature deaths tends to constitute a significant proportion of the total indirect costs. Late diagnosis of cervical cancer in Eswatini is the possible explanation behind the high cost associated with treatment of invasive cancer. Treatment in later stages requires extended chemo- and radiation therapy as well as longer hospitalizations and, consequently, contributes to high costs burden.

Our study estimated a total indirect cost of $5,2 million of which 67% ($3,5 million) was attributable to productivity loss due to premature mortality. To our knowledge this is the first study to demonstrate the magnitude of the economic burden of cervical cancer in Eswatini from the providers’ perspective.

The high proportion of costs attributable to premature mortality among the indirect costs has been noted in other studies [9, 14, 34, 35]. A study looking at productivity loss in 4 developing countries (Brazil, Russia, India and South Africa) ranked cervical cancer as the second highest cancer resulting to greater productivity loss in South Africa [36]. Worth noting is that cervical cancer costs were more significant in South Africa than in the rests of the countries considered. This could support the evidence that the burden of cervical cancer is heaviest in the sub-Saharan region [7].

Another Swedish study, assessing the economic burden of human papillomavirus related pre-cancers estimated the total annual costs at $124 million (€94 million) and of which $82 million (€62,2 million) were attributed to indirect costs. Of the total indirect costs, $48 million (€36 million) were attributable to premature mortality [12]. In a study undertaken in the United States of America, the researchers reported $3.3 billion costs associated with treatment of cervical cancer of which 66% ($2,2 billion) were indirect costs. Of the indirect costs 99% ($2,1 billion) were attributable to lost earnings as a result of premature mortality due to cervical cancer [37].

One of the key strengths of our study is that it highlights the significant economic burden associated with prevention, treatment and productivity loss related to cervical cancer. To our knowledge, our study has demonstrated for the first time the economic burden of cervical cancer from the societal perspective in Eswatini. We believe that the assumptions in our calculations are plausible. We followed the Eswatini standardized cancer care guidelines and expert information to inform variables/parameters considered for this analysis.

Our cost estimates for direct costs were consistent to those of other studies [11, 14]. To address the information gap, part of the clinical management data on screening and treatment was obtained from physicians with expert knowledge in the prevention and treatment of cervical intraepithelial neoplasia.

The paramount limitations were lack of national cumulative risk for mortality for women due to other causes, such that we were limited to adjust costs associated with premature mortality based on life expectancy. Also, the lack of cost index in Eswatini may to result to cost variation. However, to account for uncertainties, costs were varied against estimates that are normally used in this type of studies. Also, the lack of screening outcome data particularly histology results was another limitation. This underscores the need for organized surveillance systems for cervical cancer screening and management in Eswatini.

We also estimated transport cost and time for seeking health care based on the patients receiving follow-up treatment at Mbabane Government Hospital (National Referral Hospital). Transport cost for at least one companion per patient visit were also included. This partially demonstrated caregivers’ costs. However, the presented costs could be underestimated as the study did not estimate costs associated with care outside the hospital, including caregivers’ time, yet these have been reported to be among the main cost drivers in studies investigating other diseases in sub-Saharan Africa [38, 39].

In this study we employed the human capital approach to estimate the value of lost productivity due to time for seeking health care and premature mortality. This method is grounded in the economic theory and is based on the assumption that companies employ labor until the marginal value of an employee’s work productivity is equal either to the marginal cost of labor or to the employee’s gross wage [21]. This method discriminates individuals above retirement age and overestimates the costs of lost production because it disregards potential work replacement which eventually results in diminished production losses. Our proportion of the total annual costs constituted by indirect costs were consistent with those observed in other cost of illness studies [24, 30, 37, 40].

Our study presents the amount the country spent in 2018 on prevention, screening, management and treatment of CIN and cervical cancer from a societal perspective. We demonstrated how costs were distributed between direct and indirect costs within cervical cancer screening and management. This will provide health policy-makers new insights into where resources are spent.

The results are relevant to Eswatini, where introduction of a nonavalent (Gardasil 9) HPV vaccine (against HPV 6, 11, 16, 18, 31, 33, 45, 52 and 58) (GlaxoSmithKline and Merck & Co.) program is under consideration. To date, there have been a number of studies demonstrating that the vaccine reduces lifetime risks of cervical cancer induced by HPV types by 47% to 100% depending on age of coverage [31, 41, 42]. It is believed that implementation of organized prophylactic HPV vaccination will lead to greater amounts of resources spared which otherwise would have been used in the management of invasive cervical cancer.

The economic burden associated with prevention, management and treatment of CIN and cervical cancer in Eswatini is substantial. It is expected that this will increase significantly due to the increasing incident rate compounded by a high HIV prevalence rate among the reproductive age groups [36].

## Conclusion

Our findings provide reference for future economic evaluations for assessing HPV vaccination of both genders in Eswatini. Strategies for the prevention and early detection of pre-cervical cancer lesions and cancer should be prioritized. Introducing HPV vaccine is likely to result to public health care resource savings that are currently directed toward invasive cancer management with limited life-saving benefits.

## Supporting information

S1 Appendix Data sources. (DOCX)

## References

[1] Serrano B, Brotons M, Bosch FX, Bruni L: Epidemiology and burden of HPV-related disease. *Best Practice & Research Clinical Obstetrics & Gynaecology* 2018, 47:14–26. 10.1016/j.bpobgyn.2017.08.006 29037457

[2] Plummer M, de Martel C, Vignat J, Ferlay J, Bray F, Franceschi S: Global burden of cancers attributable to infections in 2012: a synthetic analysis. *The Lancet Global Health* 2016, 4(9):e609–e616. 10.1016/S2214-109X(16)30143-7 27470177

[3] Walboomers JM, Jacobs MV, Manos MM, Bosch FX, Kummer JA, Shah KV, et al.: Human papillomavirus is a necessary cause of invasive cervical cancer worldwide. *The Journal of pathology* 1999, 189(1):12–19. 10451482 10.1002/(SICI)1096-9896(199909)189:1<12::AID-PATH431>3.0.CO;2-F

[4] Badial RM, Dias MC, Stuqui B, Melli PPDS, Quintana SM, Bonfim CMd, et al.: Detection and genotyping of human papillomavirus (HPV) in HIV-infected women and its relationship with HPV/HIV co-infection. *Medicine (Baltimore)* 2018, 97(14):e9545–e9545. 29620669 10.1097/MD.0000000000009545 PMC5902291

[5] Vijayaraghavan A, Efrusy M, Lindeque G, Dreyer G, Santas C: PCN47 IMPACT OF HIV INFECTION ON INVASIVE CERVICAL CANCER INCIDENCE AND TREATMENT COSTS IN SOUTH AFRICAN WOMEN. *Value in Health* 2008, 11(3):A68.

[6] Bray F, Ferlay J, Soerjomataram I, Siegel RL, Torre LA, Jemal A: Global cancer statistics 2018: GLOBOCAN estimates of incidence and mortality worldwide for 36 cancers in 185 countries. *CA*: *A Cancer Journal for Clinicians* 2018. 10.3322/caac.21492 30207593

[7] Ginsburg O, Bray F, Coleman MP, Vanderpuye V, Eniu A, Kotha SR, et al.: The global burden of women’s cancers: a grand challenge in global health. *The Lancet* 2017, 389(10071):847–860. 10.1016/S0140-6736(16)31392-7 27814965 PMC6191029

[8] Luengo-Fernandez R, Leal J, Gray A, Sullivan R: Economic burden of cancer across the European Union: a population-based cost analysis. *The Lancet Oncology* 2013, 14(12):1165–1174. 10.1016/S1470-2045(13)70442-X 24131614

[9] Pearce A, Sharp L, Hanly P, Barchuk A, Bray F, de Camargo Cancela M, et al.: Productivity losses due to premature mortality from cancer in Brazil, Russia, India, China, and South Africa (BRICS): A population-based comparison. *Cancer epidemiology* 2018, 53:27–34. 10.1016/j.canep.2017.12.013 29353153

[10] de Oliveira C, Weir S, Rangrej J, Krahn MD, Mittmann N, Hoch JS, et al.: The economic burden of cancer care in Canada: a population-based cost study. *CMAJ Open* 2018, 6(1):E1–E10. 10.9778/cmajo.20170144 29301745 PMC5878959

[11] Östensson E, Fröberg M, Leval A, Hellström A-C, Bäcklund M, Zethraeus N, et al.: Cost of preventing, managing, and treating human papillomavirus (HPV)-related diseases in Sweden before the introduction of quadrivalent HPV vaccination. *PloS one* 2015, 10(9):e0139062. 10.1371/journal.pone.0139062 26398189 PMC4580320

[12] Östensson E, Silfverschiöld M, Greiff L, Asciutto C, Wennerberg J, Lydrup M-L, et al.: Correction: The economic burden of human papillomavirus-related precancers and cancers in Sweden. *PloS one* 2018, 13(7):e0200554. 10.1371/journal.pone.0200554 29985954 PMC6037381

[13] Hodgson TA, Meiners MR: Cost-of-Illness Methodology: A Guide to Current Practices and Procedures. *The Milbank Memorial Fund Quarterly Health and Society* 1982, 60(3):429–462. 6923138

[14] Ginindza TG, Sartorius B, Dlamini X, Östensson E: Cost analysis of Human Papillomavirus-related cervical diseases and genital warts in Swaziland. *PloS one* 2017, 12(5):e0177762. 10.1371/journal.pone.0177762 28531205 PMC5439687

[15] Drummond MF, Sculpher MJ, Claxton K, Stoddart GL, Torrance GW: Methods for the economic evaluation of health care programmes: Oxford university press; 2015.

[16] Östensson E, Silfverschiöld M, Greiff L, Asciutto C, Wennerberg J, Lydryp M-L, et al.: The economic burden of human papillomavirus-related precancers and cancers in Sweden. *PLoS One* 2017, 12(6):e0179520. 10.1371/journal.pone.0179520 28651012 PMC5484479

[17] Mogyorosy Z, Smith P: The main methodological issues in costing health care services: a literature review. In.; 2005.

[18] Ministry-of-Health.: Phalala Fund Annual Report. In. Eswatini; 2018.

[19] Ministry-of-Health: Sexual Reproductive Health Annual Program Report. In. Eswatini: Monitoring and Evaluation Unit; 2018.

[20] Ministry-of-Health: Eswatini Standardized Cancer Care and Guidelines. In. Eswatini: National Cancer Registry; 2020.

[21] Liljas B: How to calculate indirect costs in economic evaluations. *Pharmacoeconomics* 1998, 13(1):1–7. 10.2165/00019053-199813010-00001 10175982

[22] Östensson E, Hellström A-C, Hellman K, Gustavsson I, Gyllensten U, Wilander E, et al.: Projected cost-effectiveness of repeat high-risk human papillomavirus testing using self-collected vaginal samples in the Swedish cervical cancer screening program. *Acta Obstetricia et Gynecologica Scandinavica* 2013, 92(7):830–840. 10.1111/aogs.12143 23530870

[23] Gold M: Panel on cost-effectiveness in health and medicine. *Medical care* 1996, 34(12):DS197–DS199. 8969326

[24] Hanly P, Soerjomataram I, Sharp L: Measuring the societal burden of cancer: the cost of lost productivity due to premature cancer-related mortality in Europe. *Int J Cancer* 2015, 136(4):E136–145. 10.1002/ijc.29105 25066804

[25] Hatswell AJ, Bullement A, Briggs A, Paulden M, Stevenson MD: Probabilistic Sensitivity Analysis in Cost-Effectiveness Models: Determining Model Convergence in Cohort Models. *PharmacoEconomics* 2018, 36(12):1421–1426. 10.1007/s40273-018-0697-3 30051268

[26] Ministry-of-Health: Eswatini National Cancer Registry In. Edited by Health Mo. Eswatini; 2018.

[27] Kaye DR, Min HS, Herrel LA, Dupree JM, Ellimoottil C, Miller DC: Costs of cancer care across the disease continuum. *The oncologist* 2018, 23(7):798. 10.1634/theoncologist.2017-0481 29567821 PMC6058326

[28] Liu N, Mittmann N, Coyte PC, Hancock-Howard R, Seung SJ, Earle CC: Phase-specific healthcare costs of cervical cancer: estimates from a population-based study. *American Journal of Obstetrics and Gynecology* 2016, 214(5):615.e611–615.e611. 10.1016/j.ajog.2015.11.021 26627729

[29] Wolstenholme JL, Whynes DK: Stage-specific treatment costs for cervical cancer in the United Kingdom. *European Journal of Cancer* 1998, 34(12):1889–1893. 10.1016/s0959-8049(98)00232-9 10023311

[30] Wu Q, Jia M, Chen H, Zhang S, Liu Y, Prem K, et al.: The economic burden of cervical cancer from diagnosis to one year after final discharge in Henan Province, China: A retrospective case series study. *PloS one* 2020, 15(5):e0232129. 10.1371/journal.pone.0232129 32379783 PMC7205285

[31] Laytragoon-Lewin N, Nilsson PJ, Castro J, Gharizadeh B, Nyrén P, Glimelius B, et al.: Human papillomavirus (HPV), DNA aberrations and cell cycle progression in anal squamous cell carcinoma patients. *Anticancer research* 2007, 27(6C):4473–4479. 18214063

[32] Pisu M, Henrikson NB, Banegas MP: Costs of cancer along the care continuum: What we can expect based on recent literature. 2018, 124(21):4181–4191. 10.1002/cncr.31643 30475400

[33] Patel H, Wagner M, Bagdley D, Prabhu V, Kothari S: PIN21-ECONOMIC BURDEN OF CERVICAL SCREENING AND TREATMENT OF HPV-RELATED CERVICAL LESIONS AND CANCERS IN EUROPE. *Value in Health* 2018, 21:S224.

[34] Lipsy RJ: Assessing the short-term and long-term burden of illness in cervical cancer. *The American journal of managed care* 2008, 14(6 Suppl 1):S177–184. 18611085

[35] Insinga RP: Annual productivity costs due to cervical cancer mortality in the United States. *Women’s Health Issues* 2006, 16(5):236–242. 10.1016/j.whi.2006.06.005 17055376

[36] Justman J, Reed JB, Bicego G, Donnell D, Li K, Bock N, et al.: Swaziland HIV Incidence Measurement Survey (SHIMS): a prospective national cohort study. *The lancet HIV* 2017, 4(2):e83–e92. 10.1016/S2352-3018(16)30190-4 27863998 PMC5291824

[37] Nwankwo C, Corman SL, Shah R, Kwon Y: HSR19-102: Direct and Indirect Economic Burden of Cervical Cancer (CxCa) in the United States in 2015: A Mixed-Methods Analysis. 2019, 17(3.5):HSR19–102.

[38] Tanimura T, Jaramillo E, Weil D, Raviglione M, Lönnroth K: Financial burden for tuberculosis patients in low- and middle-income countries: a systematic review. *European Respiratory Journal* 2014, 43(6):1763–1775. 10.1183/09031936.00193413 24525439 PMC4040181

[39] Mamo G, Worku A, Lemma S, Demas T: Cost of illness of breast cancer patients on chemotherapy in Addis Ababa Public Hospitals, the case of Tikur Anbessa specialized teaching hospital-cross-sectional types of study. *Health Economics & Outcome Research*: *Open Access* 2017, 3(4):1–5.

[40] Daroudi R, Sari AA, Nahvijou A, Kalaghchi B, Najafi M, Zendehdel K: The economic burden of breast cancer in Iran. *Iranian journal of public health* 2015, 44(9):1225. 26587497 PMC4645780

[41] Ferreira M, Crespo M, Martins L, Félix A: HPV DNA detection and genotyping in 21 cases of primary invasive squamous cell carcinoma of the vagina. *Modern Pathology* 2008, 21(8):968–972. 10.1038/modpathol.2008.91 18500261

[42] Fox P, Seet J, Stebbing J, Francis N, Barton S, Strauss S, et al.: The value of anal cytology and human papillomavirus typing in the detection of anal intraepithelial neoplasia: a review of cases from an anoscopy clinic. *Sexually transmitted infections* 2005, 81(2):142–146. 10.1136/sti.2003.008318 15800092 PMC1764665

