# Supplementary material for: The economic burden of cervical cancer in Eswatini: Societal perspective
Source: PLoS One. 2021 Apr 15;16(4):e0250113. doi: 10.1371/journal.pone.0250113 (PMC8049330; doi:10.1371/journal.pone.0250113)
Supplement: S1 Appendix — (DOCX) [file pone.0250113.s001.docx]

## **S1 Appendix. Data sources**

**Prevention, management, and treatment of cervical intraepithelial neoplasia**

In Eswatini, recommendations for screening, management and treatment of cervical intraepithelial neoplasia include 1) cytology (Pap smear); 2) Visual Inspection with Acetic Acid (VIA) and 3) Colposcopy examination [[1](#_ENREF_1), [2](#_ENREF_2)]. Cytology results indicating presence of atypical squamous cells of undetermined significance (ASCUS) or Low grade squamous intraepithelial lesion (LSIL/CIN 1) are monitored through a scheduled follow-up cervical cytology tests until they are determined cleared or with persistent LSIL/CN1. Presence of atypical squamous intraepithelial lesion (ASCUS-H) or high-grade squamous intraepithelial lesion (HSIL/CIN 2/CIN 3) indicate either cone biopsy or loop electrosurgical excision procedure (LEEP) or total abdominal hysterectomy (TAH). Cryotherapy will be indicated for VIA positive results unless the woman is ineligible [[2](#_ENREF_2)].

Data on the number of cervical intraepithelial neoplasia screening (Cytology/Pap smear or VIA) conducted in Eswatini in 2018 was obtained from the sexual reproductive health program annual reports [[1](#_ENREF_1)]. The 2018 report provided information on the number and distribution of treatment procedures for VIA positive women and CIN (LEEP, TAH and cryotherapy). The burden for ASCUS-H or HSIL/CIN-2/CIN-3 was estimated based on the number of CIN treatment procedures reported (LEEP, TAH and cryotherapy). The Eswatini Standardized Cancer Care and Guidelines was used for establishing parameters used for mapping procedures [[2](#_ENREF_2)]. In addition, expert in the field were also consulted. These include medical practitioners both from private facility (The Clinic Group - Mbabane) and public (Mbabane Government Hospital - Chemotherapy Unit).

The Eswatini Standardized Cancer Care and Guidelines indicate that abnormal cervical intraepithelial test results should be treated with uttermost urgency. The guidelines indicated no treatment for women with low -grade squamous intraepithelial lesion (LSIL/CIN 1) and atypical squamous cells of undermined significance (ASCUS) but 3 months scheduled cytology test until lesion regressed to normal or there is evidence of persistent LSIL which will be indicated by positive cytology or VIA test results. As per the guidelines and in consultation with expert, we assumed that a woman with histologically confirmed presence of ASCUS-H or HSILCIN-2/CIN-3 was referred for Loop Electrosurgical Excision Procedure (LEEP) or cone biopsy or Total Abdominal Hysterectomy (TAH) then follow-up monitoring visits dependents on whether margins were completely or incompletely excised. VIA positives women were treated with cryotherapy followed by a bi-annual follow-up VIA tests.

**Treatment of cervical cancer**

The total number of incident and prevalent cervical cancer cases in 2018 were obtained from the National cancer registry. Treatment of cervical cancer is dependent on the disease stage. As per the Eswatini Standardized Cancer Care and Guideline [[2](#_ENREF_2)], we assumed that all histologically confirmed cervical cancer cases and VIA positives underwent staging procedures according to the International Federation of Gynecology and Obstetrics (FIGO) and treatment. Due to lack of data demonstrating the period between diagnosis and treatment, we assumed that all women were diagnosed and treated the same year. Treatment can be surgery (radical hysterectomy), radiotherapy and chemotherapy. In Eswatini, more than 90% of the cancer patients receive treatment through Phalala fund, a government funded scheme established to fund provision of specialized health care services to people of Eswatini that could not afford the payment [[3](#_ENREF_3)].

Generally, availability of cancer treatment remains limited in Eswatini particularly in the public sector. For radiotherapy patients are exclusively referred to South Africa (SA) whilst for chemotherapy, a majority would still be referred to SA for initiation and receive follow-up chemotherapy in country through a local private facility and a government own facility (Mbabane Government Hospital, a national referral hospital with newly established chemotherapy unit). To note is that regardless of where the treatment received, Phalala Fund facilitate both local and external referrals and is the bearer of the treatment costs. Diagnosis could be performed either at public or private hospital.

**References**

1. Eswatini: **Sexual Reproductive Health Annual Program Report**. In*.* Monitoring and Evaluation Unit: Ministry of Health; 2018.

2. Eswatini: **Eswatini Standardized Cancer Care and Guidelines**. In*.*: Ministry of Health; 2020.

3. Eswatini: **Phalala Fund Annual Report**. In*.* Ministry of Health; 2018.
